# Ensemble Modeling Reveals Threats to Pollination Services From Asynchronous Range Shifts Between *Camellia oleifera* and Its Specialized Wild Bee Pollinators

**DOI:** 10.1002/ece3.73714

**Published:** 2026-06-05

**Authors:** Linjie Chen, Youjin Hao, Xue Ruan, Siwen Liu, Peiru Shi, Bingchuan Zhang, Dunyuan Huang

**Affiliations:** ^1^ Key Laboratory of Pollinator Resources Conservation and Utilization of the Upper Yangtze River, Ministry of Agriculture and Rural Affairs Chongqing China; ^2^ Chongqing Key Laboratory of Vector Insects, Chongqing Normal University Chongqing China

**Keywords:** *Camellia oleifera*, climate change, habitat overlap, plant‐pollinator interactions, pollination services, species distribution models

## Abstract

*Camellia oleifera* is a self‐incompatible woody oil crop endemic to China that relies solely on insect pollination for seed production. Its flowering period, from late October to late December, coincides with seasonally low temperatures that suppress pollinator activity. Two oligolectic bee species, *Colletes gigas* and *Andrena camellia* , are *Camellia oleifera*'s dominant and most efficient pollinators. This study employed ensemble species distribution models (SSDMs) to identify the potential distributions of *C. oleifera* and these two key pollinators under current and future climate scenarios. We integrated field survey data with open biodiversity database records to simulate suitable habitats and quantify their spatiotemporal overlap. Results indicate that: (1) The distribution of *C. oleifera* is primarily regulated by mean annual temperature, whereas precipitation of the driest quarter is the key constraint for both pollinator species. (2) Under current climatic conditions, a substantial overlap of 203.38 × 10^4^ km^2^ was observed, accounting for 77.47% of the plant's suitable area and 95.47% of the pollinators' suitable area. (3) Under future scenarios, the total suitable area for both bee species is projected to increase by 21.82%, with the shared area expanding by 14.45% to 232.76 × 10^4^ km^2^. (4) The geometric centroids of suitable habitats for all three species are projected to shift northwestward, indicating a general migration toward higher latitude. These findings enhance our understanding of plant‐pollinator interaction under climate change and provide actionable insights for the spatial planning of *C. oleifera* cultivation and the conserving of its key wild pollinators.

## Graphical abstract

This study employed ensemble species distribution models (SSDMs) to project the potential distributions of *C. oleifera* and these two key pollinators under current and future climate scenarios. We integrated field survey data with open biodiversity database records to simulate suitable habitats and quantify their spatiotemporal overlap.

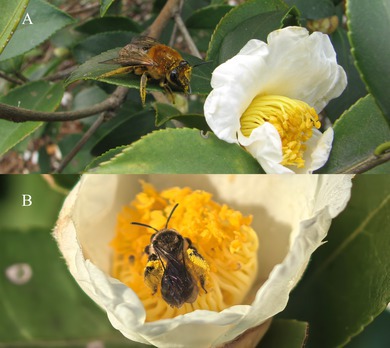

## 1 Introduction

The mutualistic relationships between flowering plants and pollinators are the product of long‐term coevolution (Nicolson and Wright 2017). Approximately 90% of flowering plant species worldwide depend on animal pollination for reproduction (Tong et al. 2023), including over 1100 of the 1300 major crop species (Lefebvre et al. 2019). Key pollinator taxa include Hymenoptera, Diptera, Coleoptera, and Lepidoptera (van der Kooi and Ollerton 2020). In return for pollination services, plants provide nutritional rewards such as pollen and nectar, which supply proteins, fats, amino acids, and vitamins essential for pollinator survival and reproduction (Di Pasquale et al. 2013). When pollinator functional diversity declines, plant‐pollinator trait matching and pollination function also decrease (Hiraiwa and Ushimaru 2024).

**FIGURE 1:**
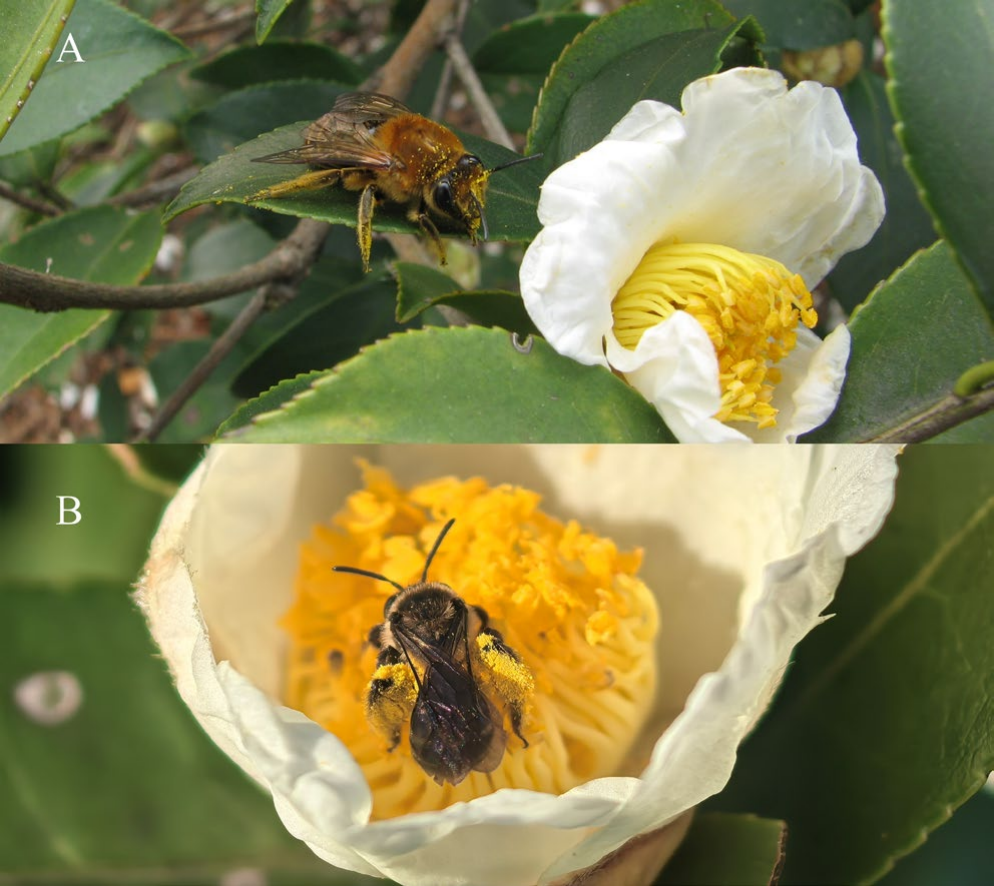
(A) *Colletes gigas* and (B) *Andrena camellia* pollinating *Camellia oleifera*. Photograph by Dunyuan Huang.

*Camellia oleifera* (Figure 1), a woody oil‐bearing species of the genus *Camellia* (family Theaceae), is endemic to China and is among the world's four major woody oil crops ( *Elaeis guineensis* , *Cocos nucifera* , and *Olea europaea* ) (Yao et al. 2005). It thrives in warm climates where it occurs on gentle slopes and acidic soils, with a natural distribution concentrated in southern China, including Hunan, Jiangxi, and Guangxi (Tu et al. 2019). As a predominantly outcrossing species exhibiting self‐incompatibility, *C. oleifera* relies heavily on insect‐pollination (Li et al. 2022). Its flowering period spans from late October to late December, a season characterized by low temperatures that naturally constrain pollinator abundance. The oligolectic bees *Colletes gigas* and *Andrena camellia* have been identified as its most effective pollinators (Figure 1), significantly outperforming the domesticated *Apis cerana* (Li, Luo, et al. 2021; Lin et al. 2023; Su et al. 2022; Zhang et al. 2022). Previous studies have established a strong positive correlation between the fruit‐set rate of *C. oleifera* and the visitation frequency of these wild bees (Li, Orr, et al. 2021). Based on flower‐visiting behavior and pollen transfer data, Huang et al. (2021) estimated that a density of approximately 2000 *A. camellia* individuals per hectare is necessary to ensure adequate pollination services. Therefore, understanding the geographic congruence between this crop and its indispensable pollinators under climate change is crucial for its sustainable cultivation.

Species distribution models (SDMs), which use species occurrence records and environmental variables to estimate ecological niches and project potential geographic distributions, have become indispensable tools in ecology (Miller 2010). Commonly employed algorithms include generalized linear models (GLM), maximum entropy models (MAXENT), and artificial neural networks (ANN), etc. However, predictions from single‐model approaches are often unstable and susceptible to sampling bias, limiting their spatial reliability (Grenouillet et al. 2011; Zhao et al. 2020). Stacked species distribution models (SSDMs), also referred to as ensemble models, integrate predictions from multiple single‐species models, often weighting them by performance (Araújo and New 2007), to account for inter‐model variability and reduce uncertainty, thereby yielding more robust and transferable predictions (Buisson et al. 2010; Fan et al. 2024). As evidenced by recent applications in biogeography, conservation planning, and invasion risk assessment, SDMs have become a valuable tool in ecological research (Bhuyan et al. 2025; Gu et al. 2023; Liu et al. 2025; Wang et al. 2024).

Current analyzes of the suitable ranges for *C. oleifera* and its two key pollinator bees indicate that *C. oleifera* , *C. gigas* , and *A. camellia* are primarily distributed in southeastern China (Jiang et al. 2025). In this study, we employ SSDMs to simulate the potential geographic distribution of *C. oleifera* and its two key wild bee pollinators ( *C. gigas* and *A. camellia* ) in China under current and future climate scenarios. Our objectives are threefold: (1) to identify the key environmental factors shaping their distributions; (2) to quantify the spatial overlap of their suitable habitats under current climatic conditions; and (3) to project how future climate change may alter habitat overlap, thereby informing pollination‐aware cultivation planning for *C. oleifera* .

## 2 Materials and Methods

### 2.1 Species Occurrence Data Collection and Preprocessing

Occurrence records for *Camellia oleifera* were obtained by querying its accepted Latin name across multiple global biodiversity databases, including the Global Biodiversity Information Facility (GBIF) (Wieczorek et al. 2012), Ecoengine, iNaturalist (iNat), and iDigBio, using the *spocc* R package (v1.2.2) (Owens et al. 2021). For the two pollinator species, *Colletes gigas* and *Andrena camellia* , distribution data were primarily compiled from our systematic field surveys, supplemented by records sourced from the published literature and the aforementioned databases. All data retrieval was completed by March 1, 2025.

**FIGURE 2:**
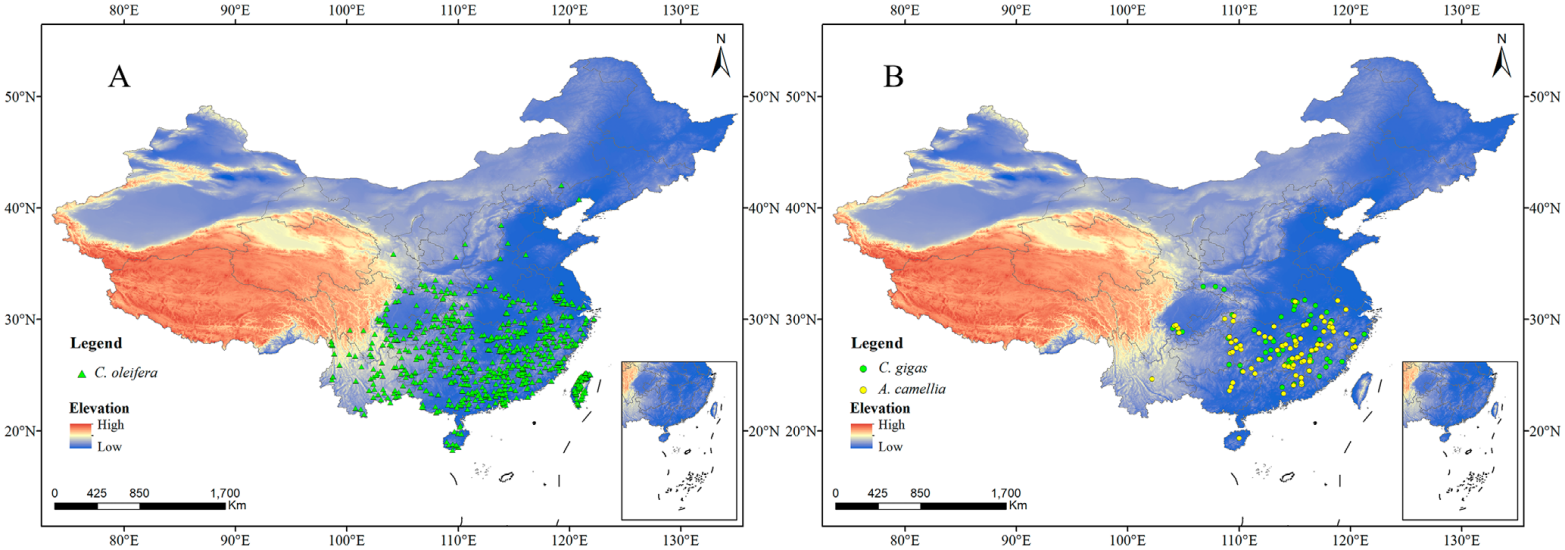
(A) Distribution data of *C. oleifera* species; (B) Distribution data of *C. gigas* and *A. camellia* [Map Review No. China GS(2019)1822].

The initial occurrence datasets were cleaned to remove spatial and temporal inaccuracies using the CoordinateCleaner R package (v3.0.1) (Zizka et al. 2019). The resulting records were then clipped to the geographical boundary of China using the Clip tool in ArcGIS 10.8. To reduce the effects of spatial autocorrelation and sampling bias, we applied spatially rarefaction using the “Spatially Rarefy Occurrence Data for SDMs” tool in SDM Toolbox (v2.5) (Brown 2014), retaining only one occurrence record per 5 arc‐minutes grid cell. This process yielded final datasets comprising 793 records for *C. oleifera* , 95 for *C. gigas* , and 82 for *A. camellia* (Figure 2, Table S1).

### 2.2 Environmental Variables and Preprocessing

To model the potential distributions of *C. oleifera* and its two dominant wild bee pollinators under current and future climate scenarios, we included 19 bioclimatic variables listed in Table 1. These variables follow standard WorldClim definitions, with data accessed via the Paleoclim database, and represent annual trends, seasonality, and extreme climatic factors derived from monthly temperature and precipitation data. Current climate data (representing the period 1979–2013) were obtained from the Anthropocene v1.2b dataset. Future climate projection (2071–2100) were derived from the MPI‐ESM1‐2‐HR climate model under the SSP370 pathway, sourced from the Coupled Model Intercomparison Project Phase 6 (CMIP6) (Jägermeyr et al. 2021; Riahi et al. 2017), and were accessed through the Paleoclim database (http://www.paleoclim.org).

**TABLE 1:** Bioclimatic variables initially considered for SSDM modeling.

Environmental factors	Description	Used (√) or Not (×)
bio1	Annual mean temperature (°C)	√
bio2	Mean diurnal range (°C)	√
bio3	Isothermality (bio2/bio7)	√
bio4	Temperature seasonality (standard deviation)	**×**
bio5	Max temperature of warmest month (°C)	√
bio6	Min temperature of coldest month (°C)	**×**
bio7	Temperature annual range (°C)	√
bio8	Mean temperature of wettest quarter (°C)	**×**
bio9	Mean temperature of driest quarter (°C)	**×**
bio10	Mean temperature of warmest quarter (°C)	**×**
bio11	Mean temperature of coldest quarter (°C)	**×**
bio12	Annual precipitation (mm/year)	√
bio13	Precipitation of wettest month (mm/month)	**×**
bio14	Precipitation of driest month (mm/month)	**×**
bio15	Precipitation seasonality (coefficient of variation)	√
bio16	Precipitation of wettest quarter (mm/quarter)	**×**
bio17	Precipitation of driest quarter (mm/quarter)	√
bio18	Precipitation of warmest quarter (mm/quarter)	**×**
bio19	Precipitation of coldest quarter (mm/quarter)	**×**

*Note:* Bioclimatic variables were screened based on annual mean temperature (bio1) and annual precipitation (bio12), only those with a Pearson correlation coefficient |*r*| < 0.85 and clear ecological significance were retained.

All environmental variables, whose definitions and units are provided in Table 1, were uniformly processed in ArcGIS v10.8 (https://desktop.arcgis.com). The data were projected to the GCS_WGS_1984 geographic coordinate system, resampled to a spatial resolution of 2.5 arc‐minutes (~5 km), and clipped to the geographical boundary of China (China GS (2019) 1822 base map).

To avoid model overfitting caused by multicollinearity among predictors, we assessed pairwise correlations among the 19 bioclimatic variables using the “Correlations and Summary Stats” tool in the SDM Toolbox. Variable pairs exhibiting a Pearson correlation coefficient |*r*| ≥ 0.85 were considered highly collinear. From each correlated pair, we retained the variable with greater ecological interpretability for the study species. Given that many bioclimatic variables are derived from mean annual temperature (bio1) and annual precipitation (bio12), these two were prioritized as reference variables. This screening process resulted in the selection of eight weak correlations |*r*| < 0.85 and clear ecological relevance for subsequent modeling: bio1, bio2, bio3, bio5, bio7, bio12, bio15, and bio17 (Table 1).

### 2.3 Ensemble Model Construction and Evaluation

Ensemble species distribution models were built using the *ssdm* package (v0.2.9) in R (Schmitt et al. 2017) to integrate multiple algorithms and produce robust predictions. Model performance was preliminarily evaluated using the Area Under the Receiver Operating Characteristic Curve (AUC), True Skill Statistic (TSS), and Kappa statistic, and only well‐performing models were retained for ensemble construction. The final ensemble incorporated seven distinct algorithms: Generalized Linear Model (GLM), Generalized Additive Model (GAM), Multiple Adaptive Regression Splines (MARS), Classification Tree Analysis (CTA), Random Forest (RF), Artificial Neural Network (ANN), and Support Vector Machine (SVM). These models represent different modeling approaches. The MaxEnt and Generalized Boosted Models (GBM) were excluded from the ensemble based on their comparatively lower predictive performance in preliminary tests.

From each model run, pseudo‐absence points were automatically generated using the R package *ssdm* (v0.2.9) (Barbet‐Massin et al. 2012; Schmitt et al. 2017). The pseudo‐absence to presence ratios were approximately 1:1.1, 1:5.2, and 1:5.9 for *C. oleifera* , *C. gigas* , and *A. camellia* , respectively. The species occurrence data were randomly partitioned, with 75% of records allocated for modeling training and the remaining 25% reserved for independent testing. Model performance was rigorously assessed through 10‐fold cross‐validation using a holdout strategy to ensure independence between the training and testing datasets (Wisz et al. 2008).

To qualify for inclusion in the final ensemble, individual models were required to achieve a minimum AUC value of 0.75. A final continuous habitat suitability map, representing the probability of occurrence, was generated by averaging projections across 101 bootstrap replicates. The relative contribution of each environmental variable to the ensemble model was estimated using the built‐in functions of the *ssdm* package. The ensemble prediction was derived by weighting the projections of the constituent models based on a composite evaluation metric integrating TSS, Kappa, and AUC values (Chau et al. 2023). Model performance was interpreted according to established thresholds (Lu et al. 2024).

To further elucidate the relationship between species presence and key environmental variables, response curves were generated using MaxEnt (v3.4.4) (Phillips et al. 2006). The model was configured with the following settings: create response curves, jackknife test for variable importance, random seed enabled, and plot data output. Key parameters were set as follows: 25% of data withheld for testing, a maximum of 10,000 background points, 2000 maximum iterations and 10 bootstrap replicates. All other parameters were retained their default values.

### 2.4 Analysis of Habitat Suitability and Spatiotemporal Distribution Shifts

The continuous habitat suitability projections generated by the ensemble SDMs were classified into three distinct suitability categories (unsuitable, low suitability, and high suitability) using the natural breaks (Jenks) method in ArcGIS. To quantify spatiotemporal changes in habitats, we calculated the geometric centroid for the combined high and low suitability areas for each species in both the current (1979–2013) and future (2071–2100) periods. The centroid locations were computed using the Region Analysis tool in ArcGIS and the *foreign* package (v0.8) in R (https://cran.r‐project.org/package=foreign). The migration distance and direction of these centroids between periods were then quantified using the *geosphere* package (v1.5) in R (https://cran.r‐project.org/package=geosphere), which calculates great‐circle distances based on spherical geometry. This centroid‐based approach provides a synthesized metric for visualizing the overall trajectory of range expansion or contraction.

## 3 Results

### 3.1 Model Performance Evaluation

**FIGURE 3:**
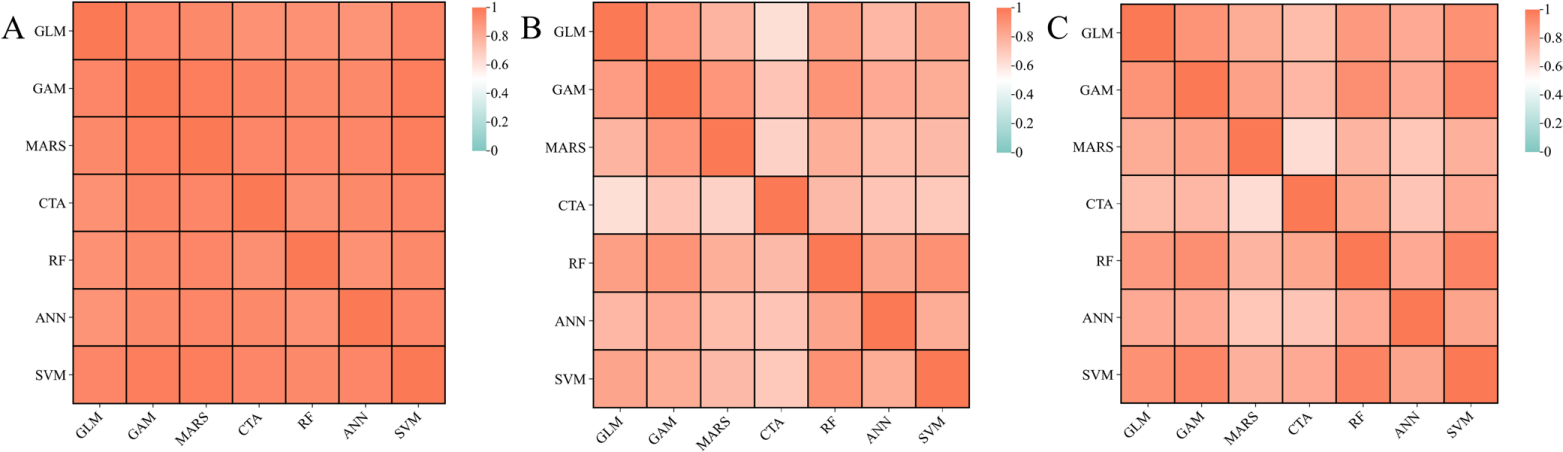
Correlation among different modeling algorithms for (A) *C. oleifera* , (B) *C. gigas* , and (C) *A. camellia* .

The ensemble model exhibited robust predictive performance for all three species under current climatic conditions (Figure 3, Table 2). Evaluation of the seven constituent algorithms revealed high predictive consistency for *C. oleifera* (correlation range: 0.90–0.98). In contrast, the pollinator species demonstrated greater variability, with correlation ranges of 0.62–0.91 for *C. gigas* and 0.63–0.96 for *A. camellia* .

**TABLE 2:** Performance evaluation of the species distribution models (SDMs) for each species under current climatic conditions.

Species	AUC	Sensitivity	Specificity	Kappa	TSS
*C. oleifera*	0.91	0.86	0.83	0.70	0.70
*C. gigas*	0.93	0.90	0.88	0.67	0.78
*A. camellia*	0.92	0.89	0.88	0.64	0.76

The integrated ensemble model achieved exceptional discriminative capacity, with AUC values of 0.91 for *C. oleifera*, 0.93 for *C. gigas* , and 0.92 for *A. camellia* . Model accuracy was further confirmed by TSS values of 0.70, 0.78, and 0.76, and Kappa coefficients of 0.70, 0.67, and 0.64, for *C. oleifera* , *C. gigas* , and *A. camellia* , respectively (Table 2). Model reliability was further corroborated by high sensitivity and specificity scores, which were 0.83 for *C. oleifera* and 0.88 for both pollinator species. Collectively, these comprehensive performance metrics demonstrated that our ensemble modeling framework provides a statistically robust and biologically meaningful representation of the potential geographic distributions for *C. oleifera* and its key wild bee pollinators. The model's strong predictive capability across multiple evaluation criteria suggests its utility for both ecological research and conservation planning applications.

### 3.2 Importance of Environmental Variables

**FIGURE 4:**
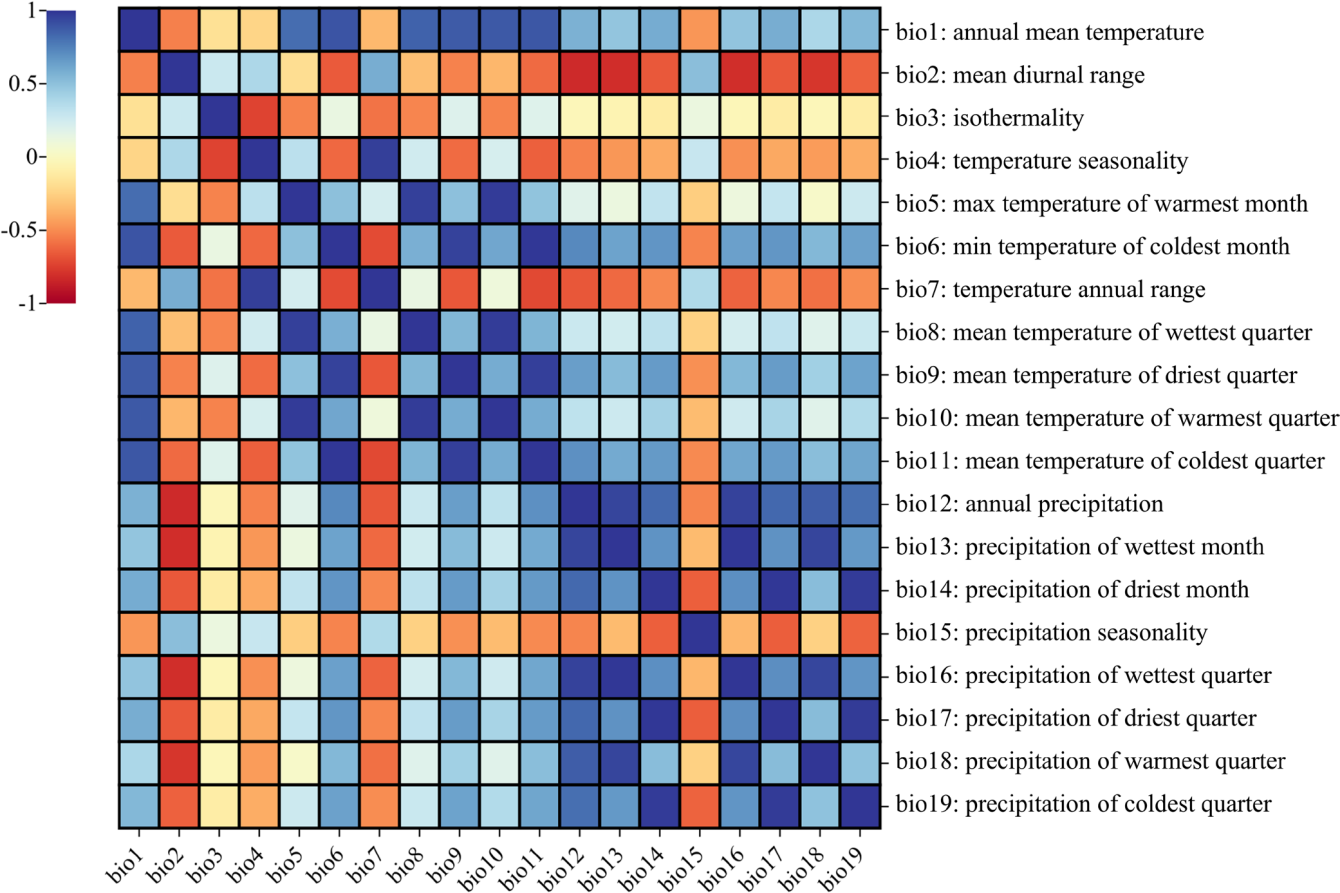
Correlations Among 19 Environmental Factors.

Analysis of the 19 initial bioclimatic variables revealed substantial pairwise correlations, ranging from −0.83 to 1.0 (Figure 4). Following variable selection, eight low‐correlation predictors (bio1, bio2, bio3, bio5, bio7, bio12, bio15, bio17) were retained, each exhibiting distinct importance patterns (Appendix Table 1, Figure 4). Under current climatic conditions, the distribution of *C. oleifera* was predominantly governed by temperature‐related factors. The four most important variables were annual mean temperature (bio1, 24.28%), temperature annual range (bio7, 14.39%), precipitation seasonality (bio15, 13.77%), and the max temperature of warmest month (bio5, 13.20%), collectively underscoring the dominant role of thermal regimes.

**FIGURE 5:**
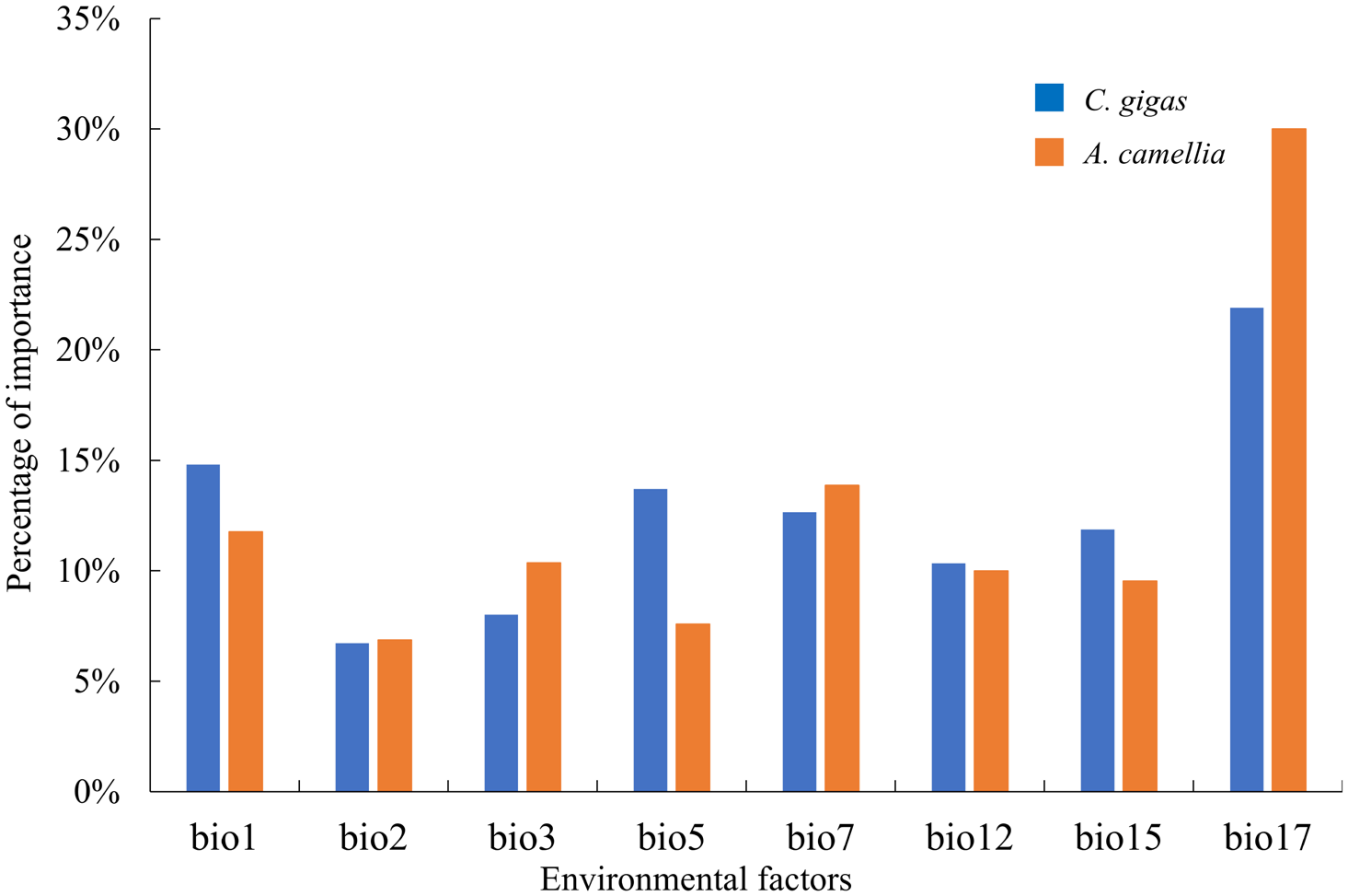
Contribution rates of environmental factors affecting *C. gigas* , and *A. camellia* .

For the pollinator *C. gigas* , distributional constraints were shaped by both precipitation and temperature variables, with the most critical predictors being precipitation of the driest quarter (bio17, 21.90%), bio1 (14.81%), bio5 (13.71%), and bio7 (12.65%). Similarliy, the distribution of *A. camellia* was primarily determined by bio17 (30.00%), bio7 (13.87%), and bio1 (11.77%). Among these, bio17 (precipitation of the driest quarter), as a key factor, plays a dominant role for both wild bee species (Figure 5, Appendix Table 1), highlighting the ecological significance of dry‐season precipitation in determining habitat suitability for these key pollinators of *C. oleifera* .

### 3.3 Overlap of Suitable Habitats Under Current Climate Conditions

**FIGURE 6:**
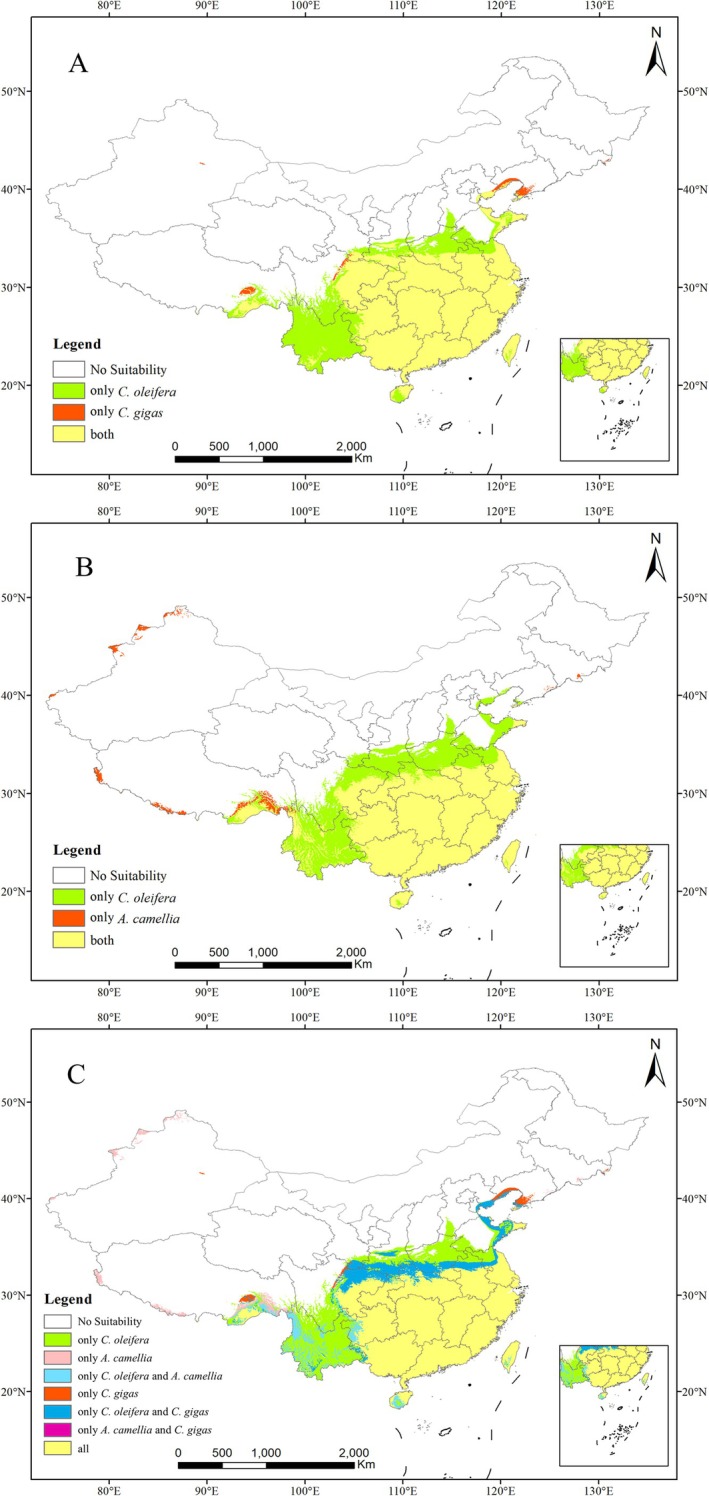
Current period distribution of suitable areas for (A) *C. oleifera* and *C. gigas* ; (B) *C. oleifera* and *A. camellia* ; (C) *C. oleifera* , *C. gigas* , and *A. camellia* [Map Review No. China GS(2019)1822].

Under current climate scenarios, the potentially suitable habitats for *C. oleifera* and its two dominant wild bee pollinators are primarily concentrated in southeastern China (Figure 6, Table 3). Among these three species, *C. oleifera* exhibits the broadest potential distribution, while *A. camellia* shows the most restricted range.

**TABLE 3:** Suitable areas for different species and their overlapping areas under current climate conditions.

Suitability			Suitability overlap		
Species	Area/(×10^4^ km^2^)	Percent/(%)	Species	Area/(×10^4^ km^2^)	Percent/(%)
*C. oleifera*	262.53	27.35%	*C. oleifera* and *C. gigas*	190.73	98.35%
*C. gigas*	193.93	20.20%			72.65%
*A. camellia*	181.44	18.90%	*C. oleifera* and *A. camellia*	174.98	96.44%
*C. oleifera* + *C. gigas*	265.73	27.68%			66.65%
*C. oleifera* + *A. camellia*	268.99	28.02%	*C. oleifera* and Bee	203.38	95.47%
*C. gigas* + *A. camellia*	213.03	22.19%			77.47%

The suitable habitat for *C. oleifera* spans 18°–35° N and 93°–123° E, covering approximately 262.53 × 10^4^ km^2^ (27.35% of China's total land area). In contrast, *C. gigas* occupies a more constrained range (19°–34° N, 104°–124° E), with an estimated suitable area of 193.93 × 10^4^ km^2^ (20.20% of China's land area). Notably, 72.65% (190.73 × 10^4^ km^2^) of *C. gigas* suitable habitat overlaps with that of *C. oleifera* , suggesting that the host plant provides sufficient floral resources for this pollinator across most of its range. However, a notable pollination services gap exists in regions such as Yunnan, the Yunnan‐Sichuan border, and the Qinling‐Huaihe line, where *C. gigas* is absent despite the presence of suitable *C. oleifera* habitat (Figure 6A).

The distribution of *A. camellia* is even more limited (18°–33° N, 104°–123° E), covering 181.44 × 10^4^ km^2^ (18.90% of China's land area). Its distribution overlap with *A. camellia* is 96.44% (174.98 × 10^4^ km^2^), representing 66.65% of the host plant's suitable area, indicating that *C. oleifera* also supports *A. camellia* effectively. Similar to *C. gigas* , *A. camellia* is also absent from key *C. oleifera* cultivation zones, including Yunnan, central Sichuan, the Qinling‐Huaihe region, and coastal Shandong (Figure 6B).

Collectively, the combined suitable area for both pollinator species covers 213.03 × 10^4^ km^2^ (22.19% of China's total area), overlapping with 95.47% (203.38 × 10^4^ km^2^) of the *C. oleifera* suitable areas (77.47% of the crop's habitat) (Figure 6C, Table 3). These results demonstrated that, under the current climate conditions, *C. oleifera* generally sustains its primary pollinators across most of its distribution. However, spatial mismatches, where the crop is present but pollinators are scarce, highlight potential pollination service limitation in several ecological and agriculturally significant regions.

### 3.4 Projected Overlap of Species Habitats Under Future Climate Scenarios

**FIGURE 7:**
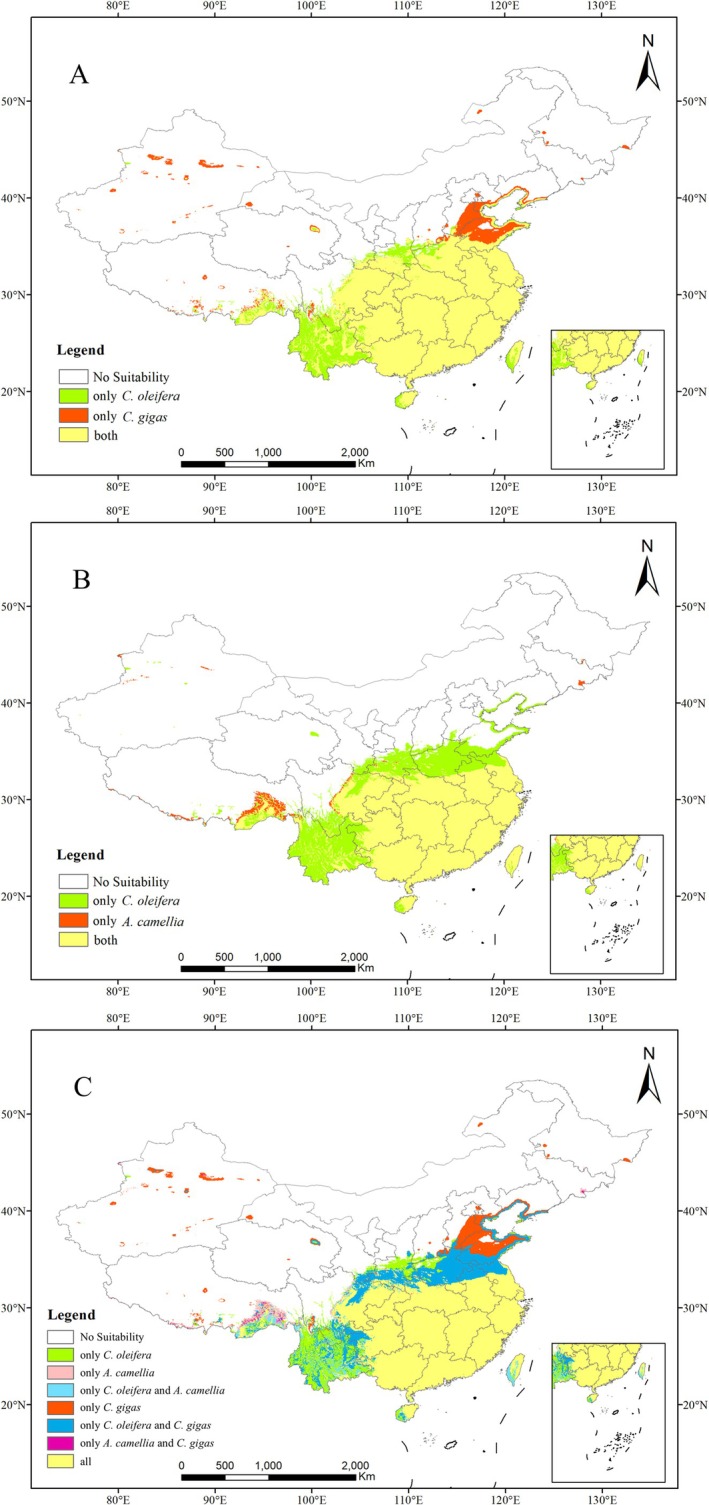
Future distribution of suitable areas for (A) *C. oleifera* and *C. gigas* ; (B) *C. oleifera* and *A. camellia* ; (C) *C. oleifera* , *C. gigas* , and *A. camellia* [Map Review No. China GS(2019)1822].

Under future climate scenarios (2071–2100), our projection reveals divergent range shifts for *C. oleifera* and its two dominant wild bee pollinators (Figure 7, Table 4). While *C. oleifera* maintains remarkable habitat stability with only a marginal 0.74% range expansion (264.47 × 10^4^ km^2^), both pollinator species exhibit substantial distributional change. The most dramatic shift occurs in *C. gigas* , projected to expand its suitable habitat by 26.99% (246.27 × 10^4^ km^2^), with notable northward colonization into previously marginal areas including southern Shandong and the Shandong‐Hebei border. This expansion enhances spatial congruence with *C. oleifera*, increasing their overlapping range to 224.75 × 10^4^ km^2^ (91.26% of pollinator habitat; 84.98% of plant habitat), a 17.84% increase from current conditions. However, persistent spatial mismatch remains evident in Yunnan, southern Taiwan, and southeastern Hainan, where *C. gigas* fails to colonize suitable *C. oleifera* habitats (Figure 7A).

**TABLE 4:** Suitable areas for different species and their overlapping areas under future climate scenarios.

Suitability			Suitability overlap			
Species	Area/(×10^4^ km^2^)	Growth rate/(%)	Species	Area/(×10^4^ km^2^)	Growth rate/(%)	Percent/(%)
*C. oleifera*	264.47	0.74%	*C. oleifera* and *C. gigas*	224.75	17.84%	91.26%
*C. gigas*	246.27	26.99%				84.98%
*A. camellia*	188.4	3.84%	*C. oleifera* and *A. camellia*	182.04	4.04%	96.62%
*C. oleifera* + *C. gigas*	285.99	7.62%				68.83%
*C. oleifera* + *A. camellia*	270.83	0.68%	*C. oleifera* and Bee	232.76	14.45%	89.70%
*C. gigas* + *A. camellia*	259.49	21.82%				88.01%

*A. camellia* shows more modest changes, with a 3.84% range increase (188.40 × 10^4^ km^2^). The species maintains exceptionally high overlap with *C. oleifera* (96.62%, 182.04 × 10^4^ km^2^), covering 68.83% of the host plant's range. It represents a 4.04% increase in overlapping area, suggesting that *C. oleifera* will continue to provide adequate floral resources for this species. Despite this, continued absence from critical cultivation zones (Yunnan, southeastern Hainan, and the Qinling‐Huaihe region) suggests enduring pollination service deficits in these areas (Figure 7B).

Our integrated projection reveals a substantial 21.82% expansion (259.49 × 10^4^ km^2^) in the combined suitable habitat area for both key wild bee pollinators under future climate scenarios. Notably, the spatial congruence between pollinator distributions and *C. oleifera* habitat is projected to increase significantly, with 232.76 × 10^4^ km^2^ (88.01% of the plant's future suitable range) showing co‐occurrence potential—representing a 14.45% increase in shared area relative to current conditions (Figure 7C, Table 4). These findings suggest that climate change may enhance the stability of this plant‐pollinator mutualism through improved habitat overlap, though the persistence of spatial mismatches in certain regions could modulate these positive effects at local scales.

### 3.5 Shifts in Habitat Centroid Under Climate Change

**FIGURE 8:**
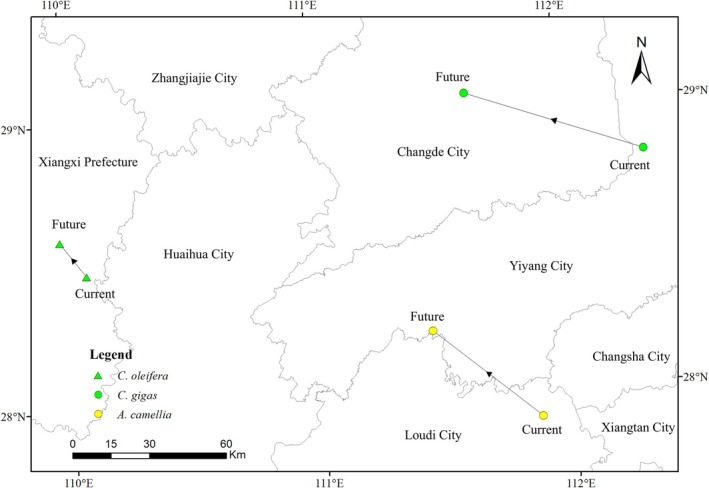
Centroid shifts of suitable areas for different species under future climate scenarios. [Map Review No. China GS(2019)1822].

Our analysis revealed marked interspecific differences in habitat centroid displacements under projected climate change scenarios (Figure 8). *C. oleifera* exhibited minimal range shifted, with its centroid moving just 16.72 km northwestward from Xiangxi Prefecture (28.48° N, 110.07° E) to a new location (28.60° N, 109.97° E). In contrast, both pollinator species showed substantially greater shifts: *C. gigas* shifted 73.2 km northwest from Yiyang City (28.81° N, 112.34° E) to Changde City (29.04° N, 111.63° E), while *A. camellia* moved 54.2 km in the same direction from Loudi City (27.90° N, 111.87° E) to Yiyang City (28.22° N, 111.45° E).

## 4 Discussion

### 4.1 Key Environmental Drivers of Distributions

Mean annual temperature (bio1) emerged as the predominant factor governing the distribution of *C. oleifera* , accounting for 24.28% to the model's predictive contribution. This pronounced thermal dependence aligns with the species' subtropical adaptation, as low temperatures can impair physiological processes, including photosynthetic efficiency, delay flowering, reduce pollen viability, and ultimately reduce fruit set (Wu 2020). In contrast, precipitation during the driest quarter (bio17) was identified as the most critical variable for both wild bee pollinators, contributing 21.9% for *C. gigas* and 30.0% for *A. camellia* . This pattern likely reflects the critical role of winter moisture availability in regulating adult bee activity, particularly during their primary foraging periods ( *C. gigas* : mid‐November to mid‐December; *A. camellia* : mid‐October to mid‐December) (Huang et al. 2008, 2015). Insufficient precipitation can compromise soil conditions for nest construction (Harmon‐Threatt 2020), whereas excessive rainfall may restrict forage activity (Dang and Chen 2011), disrupt pollen adhesion, and impair reproductive success (Chang et al. 2008). Response curve analysis further indicated optimal bio17 ranges of 107.4–269.2 mm/quarter for *C. gigas* and 107.83–273.77 mm/quarter for *A. camellia* (Appendix Table 1), highlighting the narrow hydrological thresholds required to sustain viable pollinator populations.

### 4.2 Shift in Habitat Suitability and Overlap

The current climatically suitable habitats for *C. oleifera* are primarily located south of the Qinling‐Huaihe line and east of the Tibetan Plateau, which aligns with previous reports (Wang et al. 2020). Under future climate scenarios, our models predict a moderate expansion of suitable areas, with a northwestward shift in its distribution centroid. Similarly, the suitable habitat of *C. gigas* and *A. camellia* is also expected to expand, with their distribution centroids shifting northward. Notably, our projected distribution for *A. camellia* exhibits greater spatial extent than that reported by Zhang (2023), a discrepancy that may stem from differences in occurrence records and environmental variable selection.

Currently, over 95% of the suitable habitat for these two dominant wild bee pollinators overlaps with that of *C. oleifera*. However, these shared areas represent less than 80% of the plant's total suitable area, suggesting that while *C. oleifera* provides adequate floral resources to sustain pollinator populations, the spatial coverage of pollination service does not currently match the full extent of the plant's distribution. By the future period, the overlap is projected to increase to over 88% of the plant's suitable area, reflecting stronger geographic coupling between the mutualists and potentially more stable plant‐pollinator interactions under climate change.

Such changes in spatial coupling are consistent with previous studies showing that climate change can alter plant‐pollinator interactions (Freimuth et al. 2022). In some cases, climate change may enhance spatial overlap between interacting species, thereby strengthening ecological interactions. In other cases, however, it may lead to temporal or spatial mismatches that disrupt these relationships. For example, the decreased phenological synchronization between bumble bees and flowering plants can arise from differential responses to climate change (Pyke et al. 2016), whereas reduced future overlap between *Agave* species and their endangered pollinating bat may result in spatial decoupling (Gómez‐Ruiz and Lacher 2019).

### 4.3 Implications for Cultivation Management

Currently *C. oleifera* cultivation suffers from the “thousand flowers, one fruit” phenomenon, a well‐documented yield limitation primarily caused by inadequate pollination services (Yuan et al. 2023). Our analysis identifies a significant pollination gap, with over 22% of the suitable *C. oleifera* area, including regions as Yunnan, southern Sichuan, and parts of the Qinling‐Huaihe region, lacking populations of the key wild pollinators. Although alternative pollinators (e.g., *Apis cerana* , hoverflies, flies) may provide partial pollination services (Cao et al. 2025; Yuan et al. 2022), they cannot match the efficiency of these specialist bees, This limitation is particularly concerning given recent findings that *C. oleifera* pollen saponins exhibit toxic effects on honeybee larvae (Zhang et al. 2024). Furthermore, the differential migration patterns of *C. oleifera* and the key pollinator bees suggest possible phenological mismatches and reduced pollination efficiency in current overlap zones, highlighting a critical climate change vulnerability for this ecological mutualism. Based on these finds, we propose a target cultivation strategy: (1) Avoiding large‐scale plantations beyond the core distribution range of *C. gigas* and *A. camellia* . (2) Prioritizing cultivation in regions demonstrating high habitat overlapping, including the eastern Sichuan Basin, Chongqing, southern Hubei, Hunan, Jiangxi, southern Anhui, southern Jiangsu, Zhejiang, Fujian, eastern Guizhou, and most parts of Guangdong and Guangxi. This spatially explicit approach would simultaneously enhance pollination efficiency and oil tea yield, conserve these oligolectic pollinators, and establish a sustainable, mutually beneficial agroecosystem. The implementation of such geographically informed management strategies could significantly improve the economic viability of *C. oleifera* cultivation while maintaining critical pollinator biodiversity.

## Author Contributions

**Linjie Chen:** conceptualization (equal), data curation (equal), formal analysis (equal), investigation (equal), software (equal), writing – original draft (equal), writing – review and editing (equal). **Youjin Hao:** writing – review and editing (equal). **Xue Ruan:** investigation (equal), writing – review and editing (equal). **Siwen Liu:** investigation (equal), writing – review and editing (equal). **Peiru Shi:** investigation (equal), writing – review and editing (equal). **Bingchuan Zhang:** funding acquisition (equal), project administration (equal), resources (equal), supervision (equal), writing – review and editing (equal). **Dunyuan Huang:** funding acquisition (equal), investigation (equal), methodology (equal), project administration (equal), resources (equal), supervision (equal), writing – review and editing (equal).

## Funding

This work was supported by the Natural Science Foundation of Chongqing, China (CSTB2022 NSCQ‐MSX0985), the National Natural Science Foundation of China (31970484), and the Chongqing Modern Agricultural Industrial System, China (CQMAITS202516).

## Conflicts of Interest

The authors declare no conflicts of interest.

## Supporting information


**Appendix Table 1.** Distribution site data for each species.

**Data S1:** ece373714‐sup‐0002‐AppS1.docx.

## Data Availability

The data that support the findings of this study are in the main text and available in the Appendix S1 of this article.
