# Supplementary material for: Ensemble Modeling Reveals Threats to Pollination Services From Asynchronous Range Shifts Between Camellia oleifera and Its Specialized Wild Bee Pollinators
Source: Ecol Evol. 2026 Jun 5;16(6):e73714. doi: 10.1002/ece3.73714 (PMC13238709; doi:10.1002/ece3.73714)
Supplement: Supplementary file 2 — Data S1: ece373714‐sup‐0002‐AppS1.docx. [file ECE3-16-e73714-s001.docx]

**APPENDIX TABLE 1** **|** Variables used in modeling and their contribution to the model.

| Environmental factors | *Collects gigas* | | *Andrena camellia* | | *Camellia oleifera* | | Unite |
| --- | --- | --- | --- | --- | --- | --- | --- |
|  | Percentage of importance | High suitability range | Percentage of importance | High suitability range | Percentage of importance | High suitability range |  |
| bio1 | 14.81% | 17.31-20.89 | 11.77% | 17.28-20.85 | 24.28% | 18.27-23.60 | °C |
| bio2 | 6.72% | 6.12-7.59 | 6.87% | 6.08-7.32 | 12.20% | 2.45-6.07 | °C |
| bio3 | 8.01% | 0.20-0.25 | 10.36% | 0.21-0.24 | 8.21% | 0.21-0.24 | bio2/bio_7 |
| bio5 | 13.71% | 31.40-43.08 | 7.59% | 31.11-34.04 | 13.20% | 30.56-33.22 | °C |
| bio7 | 12.65% | 26.95-31.92 | 13.87% | 26.53-31.41 | 14.39% | 6.74-19.93 | °C |
| bio12 | 10.34% | 1301.04-1953.74 | 9.99% | 1271.68-2093.17 | 8.33% | 1394.02-3931.77 | mm/year |
| bio15 | 11.87% | 39.66-60.77 | 9.54% | 39.68-59.55 | 13.77% | 3.3-63.62 | coefficient of variation |
| bio17 | 21.90% | 107.40-269.20 | 30.00% | 107.83-273.77 | 5.62% | >97.16 | mm/quarter |
